# The quality of lymph node harvests in extralevator abdominoperineal excisions

**DOI:** 10.1186/s12893-020-00898-2

**Published:** 2020-10-16

**Authors:** Ben Liu, Ja’Quay Farquharson

**Affiliations:** Department of General Surgery, New Cross Hospital, The Royal Wolverhampton NHS Trust, Wolverhampton Road, Wolverhampton, WV10 0QP West Midlands UK

**Keywords:** Rectal cancers, Abdominoperineal excisions, Lymph nodes

## Abstract

**Background:**

Lymph node (LN) harvest in colorectal cancer resections is a well-recognised prognostic factor for disease staging and determining survival, particularly for node-negative (N0) diseases. Extralevator abdominoperineal excisions (ELAPE) aim to prevent “waisting” that occurs during conventional abdominoperineal resections (APR) for low rectal cancers, and reducing circumferential resection margin (CRM) infiltration rate. Our study investigates whether ELAPE may also improve the quality of LN harvests, addressing gaps in the literature.

**Methods:**

This retrospective observational study reviewed 2 sets of 30 consecutive APRs before and after the adoption of ELAPE in our unit. The primary outcomes are the total LN counts and rates of meeting the standard of 12-minimum, particularly for those with node-negative disease. The secondary outcomes are the CRM involvement rates. Baseline characteristics including age, sex, laparoscopic or open surgery and the use of neoadjuvant chemoradiotherapy were accounted for in our analyses.

**Results:**

Median LN counts were slightly higher in the ELAPE group (16.5 vs. 15). Specimens failing the minimum 12-LN requirements were almost significantly fewer in the ELAPE group (OR 0.456, P = 0.085). Among node-negative rectal cancers, significantly fewer resections failed the 12-LN standard in the ELAPE group than APR group (OR 0.211, P = 0.044). ELAPE led to a near-significant decrease in CRM involvement (OR 0.365, P = 0.088). These improvements were persistently observed after taking into account baselines and potential confounders in regression analyses.

**Conclusion:**

ELAPE provides higher quality of LN harvests that meet the 12-minimal requirements than conventional APR, particularly in node-negative rectal cancers. The superiority is independent of potential confounding factors, and may implicate better clinical outcomes.

## Background

Abdominoperineal resections (APR) are the established curative surgical treatment for low rectal cancers within 4 cm from the anal verge [1]. A high rate of intraoperative bowel perforation (IBP) and risks of positive circumferential resection margin (CRM), both strong predictors of survival [2] had been reported to be as high as 30.4% in the Dutch TME trial [3] and 30.2% in the MERCURY trial [4]. These have subsequently been correlated with higher recurrence rates and reduced survival after APR [5]. Extralevator Abdominoperineal Excision (ELAPE) had been described to standardise a cylindrical specimen without a “waist” in order to minimise CRM involvement, and early outcomes have been favourable [6–8]

In addition to CRM, the identification of lymph node (LN) metastases following surgical resection for colon and rectal cancer is well recognised as a key prognostic factor [9], and is a pre-requisite in accurate cancer staging [10]. Established evidence had demonstrated strong association between higher total LN counts and improved disease survival [11, 12] particularly for node negative colorectal cancers [12, 13]. The presence of LN metastasis determines those most likely to benefit from adjuvant therapies as shown in multiple key phase III trials including the MOSAIC trial [14]. Although there are still debates regarding the optimal number of LNs required for adequate staging [15], the evaluation of at least 12 LNs following colorectal resection is widely recommended in most clinical guidelines [16, 17]

It is not well understood how ELAPE may have an impact on recurrences and disease survival. The aim of our observational study therefore is to investigate differences in the number of lymph nodes yielded from ELAPE compared with conventional APR, which may be contributory to improvement in clinical outcomes. Evidence had shown diminishing returns from excessive lymph node dissection beyond the recommended 12 lymph nodes [18], our study hence focuses on whether ELAPE may be superior at meeting the 12 LN minimal requirement.

## Methods

### Study objectives

The primary objective of this observational study is to determine if ELAPE improves the number of LN yield and the success of meeting the 12LN minimal requirement, compared with standard APR. The secondary objective is to compare the CRM involvement rates and determining whether the known advantages of ELAPE can be reproduced in a district general hospital setting.

The null hypothesis is finding no differences in the pathological outcomes between the two groups (ELAPE vs. APR). We evaluate whether any significant differences are independent of patients’ baseline characteristics and other cofounders including the use of open / laparoscopic surgery and neoadjuvant chemoradiotherapy.

### Endpoints

The primary endpoints are:

- Absolute number of lymph node yields
- Failure rates to meet the 12 LN nodes pathological requirement
- Failure rates to meet the 12 LN pathological requirement in node-negative (N0) subgroups

The secondary endpoint is.

- The rates of CRM involvement

From the register of this single centre, we included patients who underwent the two techniques of abdominoperineal resections for distal (low) rectal cancers (i.e. adenocarcinoma) over a 10 year period between 2009 and 2019.

Patients undergoing revisional or completion procedures or procedures for non-adenocarcinomas were excluded.

### Data collection

Two groups of 30 consecutive cases were sampled over three years period before and after our adoption of ELAPE. Case notes were retrospectively reviewed for baseline characteristics including patients’ age and sex. The operation notes were reviewed to determine the techniques employed and the types of access (open or laparoscopic). The uses of neoadjuvant therapies were documented.

### Statistics

The UK Kingdom National Bowel Cancer Audit [17] showed a median of 15.1 LN harvested from rectal resections, and 78.6% of them achieved the 12 LN minimum. Shen et al. [19]in 2009 noted the mean number of LN ranges from 13.6 to 19; the standard deviation was 10.5. Based on this data, at the power of 80% the minimum sample size for capturing a 5% difference between the two groups is 60.

Mann–Whitney U Test was used to detect any significant differences in the absolute LN yield. Odds ratios were used to analyse differences in the rate of specimens failing the minimum 12-LN requirement between the two groups. The same is applied to rates of CRM involvement. Regression analyses were used to detect differences between the two groups independently of potential confounders (baseline characteristics, laparoscopic/open surgery and the use of neoadjuvant therapies).

Missing data were minimal as we histological data is systematically gathered through our electronic system. If any were to arise, intention to treat analyses would be performed.

## Results

**Fig. 1:**
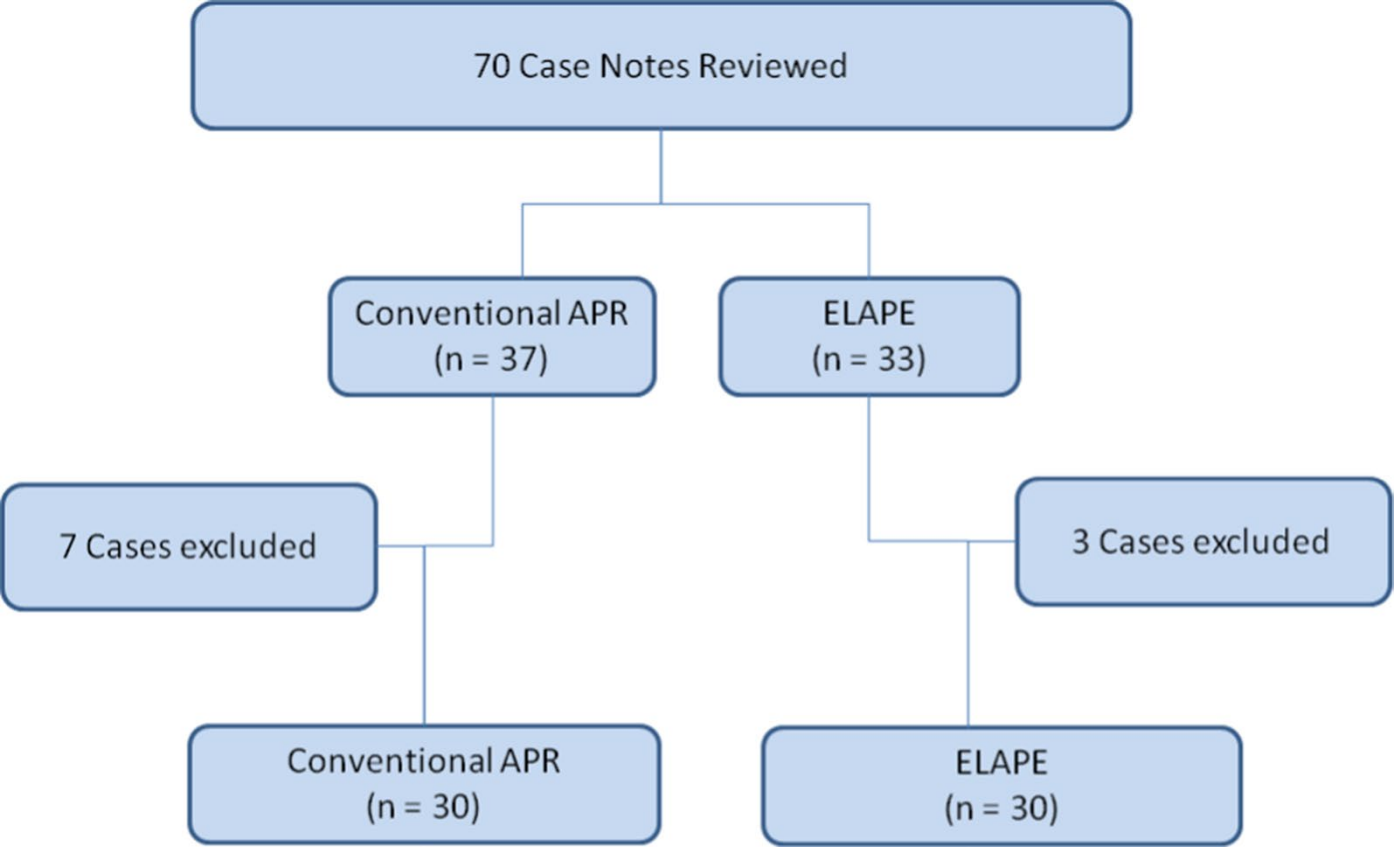
Flow chart of case selection for the ELAPE and APR groups

After excluding 7 patients from the APR group and 3 patients from the ELAPE group according to our exclusion critera, there are 30 patients per study group included in the analyses. The exclusions are described in the flow diagram (Fig. 1).

### Baseline characteristics

The baseline characteristics are shown in Table 1. There is no statistical difference in the genders and ages of patients between the two groups. Significantly fewer traditional APR cases (4/30) were performed laparoscopically compared with the ELAPE group (11/30) P = 0.037. There were no significant differences in the number of patients receiving neoadjuvant therapies between the two groups.

**Table 1:** Baseline characteristics of the ELAPE and APR groups

	Conventional APR	ELAPE	P value
Male:Female	22:8	17:13	0.176
Age	67.8	68.0	0.930
Laparoscopic:open surgery	4:26	11:19	0.037
Neoadjuvant chemoradiotherapy	14/30	16/30	0.606

### Lymph node counts

The median number of lymph node harvested from conventional APR and ELAPE specimens were 15 and 16.5 respectively (Table 2) without significant differences between them (P = 0.181). The ELAPE group has lower rates of lymph node harvests failing the minimum 12 requirement (7/30) compared with the conventional APR group (13/30), with odds ratio of 0.456 which almost reached statistical significance (P = 0.085). In those patients with negative nodal metastases status (N0), the rate of failing the 12-LN requirement is significantly reduced (3/19) in the ELAPE group compared with the APR group (8/17), with odds ratio of 0.211 (P = 0.025). When comparing the rates of pathological CRM involvement in the specimens, ELAPE (3/30) outperforms conventional APR (7/30) with odds ratio of 0.365, which almost reached statistical significance (P = 0.088).

**Table 2:** Comparisons of pathological results between the ELAPE group and APR group

	Median total lymph nodes (LN)	Below 12 LN requirement	Node negative (N0), < 12 LN requirement	CRM involvement	Total number of cases
ELAPE	16.5	7/30	3/19	3/30	30
Conventional APE	15	12/30	8/17	7/30	30
Statistical tests and significance	Mann–Whitney U Test (P = 0.181)	Odds ratio (OR) 0.456 (P = 0.085) (CI 0.149–1.396)	OR 0.211, P = 0.025) (CI 0.044–1.002)	OR 0.365, P = 0.088) (CI 0.085–1.576)	60
Regression analysis (including age, sex, lap/open, neoadjuvants)	P = 0.224	P = 0.129	P = 0.044	P = 0.099	60

### Regression analyses

There were no significant independent differences between the two groups in the median lymph node counts (P = 0.224), the rate of failure to reach 12 LN (P = 0.129) and the rates of CRM involvement (P = 0.099). However in those with nodal negative status (N0), significantly lower failure rates of harvesting 12 LN minimum were consistently observed (P = 0.044) in the ELAPE group, which is independent of the aforementioned confounders.

## Discussion

In this study we have compared lymph nodes harvests from ELAPE and conventional APR. The median number of LN yielded from conventional APR is 15, which is the same number as those reported from 69 retrospective cases by Shen et al. 2009 [19]. The media LN count of 16.5 in our ELAPE group is slightly higher than the APR group, and is comparable to the median of 13.7 nodes from a large scale (n = 519) Swedish population study on ELAPE [20]. Neither our study nor the Swedish cohorts found significant differences between APR and ELAPE groups. However, reported number of LN harvest from APRs in literature varies greatly. In general LN counts for abdominoperineal excisions are lower than other type of colorectal resections, and as low as 9 LN were reported [21]. The number of lymph nodes harvested at our unit appears higher compared to most literature. This may be explained by the contemporaneousness of our data, with higher proportions of laparoscopic surgery compared with established evidence. Dolan et al. [22], in a prospective study of 896 patients spanning 20 years (1997–2016), had found significant independent correlation between later operative date, increasing prevalence of laparoscopic surgery and higher lymph node harvests.

### 12-LN minimal requirement

Secondly, we noticed a reduction in the rate of resections failing to meet the 12-LN minimal requirement under the ELAPE technique compared with traditional APR. The differences are considerable and almost statistically significant. There is a paucity of evidence from the literature comparing the adequacy of LN harvests between abdominoperineal excision techniques despite emphases of its importance by many authors [13, 23–25] and guidelines from National Cancer Institute [16]. A minimum of 12 lymph nodes was recommended [23] as below this cut-off value there is a high risk of false-negatives in reporting lymph node metastases due to inadequate sampling [25]. The 12-node standard has been endorsed by other researches for reasons of “diminishing returns” beyond the examination of 12–17 nodes [18]. In our study, 60% and 77% of specimens in the APR and ELAPE groups respectively met this standard. When compared with results from authors who specifically investigated the 12-LN standard among rectal cancers, our cohort of patients have achieved higher rates of success in general. Field et al. [21] reported 50%, while Gurawalia et al. [26] achieved 52%, and Baxter et al. [27] had a 46.4% attainment rate. Our higher success rate could be explained again by the contemporaneousness of our cases and prevalence of laparoscopic surgery, both considered independent determinants of meeting the 12-LN standard [22]

### Node negative disease

British reviewers Ong and Schofield [28] have summarised that node-negative colorectal cancer patients have a 5-year survival rate of 70–80% in contrast to 30–60% for their nodal-positive counterparts. Survival can be improved in the latter group by adjuvant chemotherapy [14]. The 20–30% disease recurrence in apparently completely excised tumours without LN metastases is thought to be due to occult LN disease [29]. If this subset of patients could be identified by better lymph node staging, they may then benefit from adjuvant chemotherapy. Nodal-positive resections, irrespective of the number of LN harvested, would indicate adjuvant chemotherapy. Therefore substantial researches have focused on the accuracy of nodal staging and prognosis among node-negative individuals, in whom adequacy of LN harvests bears greater prognostic value [12, 30, 31]. We have similarly conducted a separate analysis on nodal-negative cases to determine whether the minimum 12-LN standard was met in this subset. We have found significantly lower rates of failures in meeting the requirement among the ELAPE group compared with APRs. This suggests that ELAPE may be superior at minimising false-negatives in apparent node-negative diseases, leading to more appropriate staging and decision-making on adjuvant treatments. The mechanism underlying our observation is not entirely understood, since anatomically the mesorectum tapers and diminishes as it adjoins the pelvic floor. However as Holms et al. eluded to in their paper [6], while other techniques (e.g. intersphincteric, extrasphincteric dissections) exist, ELAPE offers a standardised approach in abdominoperineal excisions, leading to more consistent quality of resections that in turn reduces sub-standard Total Mesorectal Excisions (TME)s.

### CRM involvement

The overall CRM rate of our cohort is 16.7%, which is relatively high but comparable to published data of 16.6% from Great Britain [32] and 16.7% from Canada [33]. Our study has showed a decreased rate of CRM involvement among the ELAPE group compared with conventional APR, though not reaching a statistically significant level. Several studies [20, 34, 35] have similarly failed to show significant superiority of ELAPE in CRM clearance.  Among them, the Danish study on ELAPE [35] suggests a magnitude of CRM+ve reduction (OR 0.386) that is comparable to our data (OR of 0.365). The more recent, yet small (n = 34) randomised controlled trial (RCT), nevertheless, demonstrated a significantly improved CRM in their ELAPE arm [36]. Our investigation suggests that this apparent benefit of ELAPE may be reproducible in a district general hospital setting among 8 colorectal surgeons at our unit.

### Potential confounders

The quality of surgery is undoubtedly a major determinant of LN harvests [37, 38]. However, other clinicopathologic factors may also influence lymph node retrievals. In particular, studies have demonstrated significant reductions in the mean LN-yields among patients who received neoadjuvant chemoradiotherapy-from 17 to 13 [39], and from 19 to 16 [40]. Other authors including Field et al. [21] have found young, female patients and higher T stages of cancers to correlate with higher LN yield. There were also significant links between laparoscopic surgery and higher rate of succeeding the 12-LN standard [22]. On this basis, we have conducted regression analyses taking into account the above predictors of LN yield as potential confounders. This has not changed the correlations found in our results. The rate of failure to achieving 12LN standard remains significantly lower in the ELAPE group among the nodal negative cohorts. The reduction in CRM involvement is still present but not statistically significant. Our analyses have suggested the superiority of ELAPE to be independent of these factors.

### Limitations

As a retrospective observational study, our investigation is subject to the usual limitations of selection and recall biases. The aim of our research was to establish whether ELAPE leads to a better pathological results. We have yet to establish whether this would necessarily translate to an improvement in clinical outcomes i.e. local recurrence rate (LR) and disease survival. Several systematic reviews on ELAPE found no improvement in either CRM or LR [34, 41]. Some mata-analyses [42] showed that despite ELAPE significantly lowering the rates of CRM involvement, it did not lead to subsequent benefits in the LR, while others [42], to the contrary, did demonstrate a significant reduction in LR (OR 0.30, P < 0.01). Even if the LR were found to be improved with ELAPE, some authors had found no difference in survival or disease progression in both a prospective study [43] a randomised controlled trial with median follow-up of 20 months [44]. However these studies have been marred by their small recruitment numbers (n = 69 and n = 67 respectively) and short follow-up periods.

Less disputable, nevertheless is evidence that a larger number of lymph nodes retrieved lead to a survival advantage [30]. This was initially attributed to upstaging cases of “missed” positive lymph nodes. However, more recent studies suggest that this phenomenon cannot be explained by staging migration alone. A systematic review [18] found improved survivals correlating with higher lymph node harvests in stage-III as well as stage-II diseases. Furthermore, lymph node sampling past a certain point does not appear to improve disease staging [45]. Interestingly Joseph et al. [46] found that better LN harvests improve cancer survival irrespective of patients’ nodal statuses (N1 or N0). Tumour-host interactions may be a plausible explanation for this, as higher LN yield may reflect a stronger host immune response [47].

Our results had lacked pathological reporting of the lengths of our specimens. This may be because there has not been a standardised length of resection for APRs in general. The amount of mesentery associated with specimen length undoubtedly correlates with the number of LNs found [19] and therefore a potential source of bias. Despite our effort to improve LN harvests, no internationally recognised standards of practice have been developed for the histopathological processing of lymph nodes in specimens [48]. A notable Canadian study showed that only 58% of pathologists were aware of current guidelines and that only 25% recognized that a minimum of 12 LNs was necessary for accurate designation of node negativity [49]. The potentially variable attentivenss and experience among out pathologists may be a source of bias. Similarly the different experiences among our 9 colorectal surgeons who perform APERS/ELAPE may also have contributed to the observed differences between the two study groups. The apparent advantages of ELAPE need to be balanced with its associated morbidity. Authors have reported significantly greater post-operative wound infections after ELAPE (20.5%) than for APR (12%) [20]. A prospective multicentred trial [50] also suggested higher rates of sexual dysfunction, urinary retention, and perineal complications associated with ELAPE. On contrary, a meta-analysis [51] found no differences in complication rates between the techniques, and some of these complications can be mitigated with reconstructions using meshes or plastic surgery. Regardless, our enthusiasm for ELAPE should always be tempered with caution and consideration of its higher complexity and potential morbidity.

## Conclusion

The findings of our comparative study has concluded that ELAPE is conducive to superior lymph node harvests particularly in regards to achieving the 12 lymph node requirement for accurate staging and appropriate adjuvant treatment of rectal cancer, and especially in apparently node negative rectal cancers. The outcome is independent of several known factors that can affect lymph node counts. A CRM+ve reduction was also deemed reproducible in a district general hospital setting. The long-term clinical outcomes in terms of recurrences and survival are still to be determined by large multicentre RCTs, which will also serve to confirm the main results of this retrospective study.

## Data Availability

The datasets used and analysed during the current study are available from the corresponding author on reasonable request.

## Abbreviations

APR: Abdominoperineal resections
CRM: Circumferential resection margin
ELAPE: Extralevator abdominoperineal excisions
IBP: Intraoperative bowel perforation
LN: Lymph node
LR: Local recurrence
RCT: Randomised controlled trial
TME: Total mesorectal excision
